# Trajectories of Frailty With Aging: Coordinated Analysis of Five Longitudinal Studies

**DOI:** 10.1093/geroni/igab059

**Published:** 2022-01-15

**Authors:** Natalie D Jenkins, Emiel O Hoogendijk, Joshua J Armstrong, Nathan A Lewis, Janice M Ranson, Judith J M Rijnhart, Tamer Ahmed, Ahmed Ghachem, Donncha S Mullin, Eva Ntanasi, Miles Welstead, Mohammad Auais, David A Bennett, Stefania Bandinelli, Matteo Cesari, Luigi Ferrucci, Simon D French, Martijn Huisman, David J Llewellyn, Nikolaos Scarmeas, Andrea M Piccinin, Scott M Hofer, Graciela Muniz-Terrera

**Affiliations:** 1 Edinburgh Dementia Prevention, University of Edinburgh, Edinburgh, UK; 2 Department of Epidemiology & Data Science, Amsterdam Public Health Research Institute, Amsterdam UMC–Location VU University Medical Center, Amsterdam, The Netherlands; 3 Department of Health Sciences, Lakehead University, Thunder Bay, Ontario, Canada; 4 Department of Psychology, University of Victoria, Victoria, British Columbia, Canada; 5 College of Medicine and Health, University of Exeter, Exeter, UK; 6 School of Rehabilitation Therapy, Queen’s University, Kingston, Ontario, Canada; 7 Research Centre on Aging, University of Sherbrooke , Sherbrooke, Québec, Canada; 8 Lothian Birth Cohorts, University of Edinburgh, Edinburgh, UK; 9 Division of Psychiatry, University of Edinburgh, Edinburgh, UK; 10 Department of Neurology, Aiginition Hospital, National and Kapodistrian University of Athens Medical School, Athens, Greece; 11 Rush Alzheimer’s Disease Center, Rush University Medical Center, Chicago, Illinois, USA; 12 Geriatric Unit, Azienda Sanitaria Toscana Centro, Florence, Italy; 13 IRCCS Istututi Clinici Scientifici Maugeri, University of Milan, Milan, Italy; 14 National Institute on Aging, Baltimore, Maryland, USA; 15 Department of Chiropractic, Macquarie University, Sydney, Australia; 16 Alan Turing Institute, London, UK; 17 Department of Neurology, Columbia University, New York City, New York, USA

**Keywords:** Age-related changes, Latent growth curve, Longitudinal

## Abstract

**Background and Objectives:**

There is an urgent need to better understand frailty and its predisposing factors. Although numerous cross-sectional studies have identified various risk and protective factors of frailty, there is a limited understanding of longitudinal frailty progression. Furthermore, discrepancies in the methodologies of these studies hamper comparability of results. Here, we use a coordinated analytical approach in 5 independent cohorts to evaluate longitudinal trajectories of frailty and the effect of 3 previously identified critical risk factors: sex, age, and education.

**Research Design and Methods:**

We derived a frailty index (FI) for 5 cohorts based on the accumulation of deficits approach. Four linear and quadratic growth curve models were fit in each cohort independently. Models were adjusted for sex/gender, age, years of education, and a sex/gender-by-age interaction term.

**Results:**

Models describing linear progression of frailty best fit the data. Annual increases in FI ranged from 0.002 in the Invecchiare in Chianti cohort to 0.009 in the Longitudinal Aging Study Amsterdam (LASA). Women had consistently higher levels of frailty than men in all cohorts, ranging from an increase in the mean FI in women from 0.014 in the Health and Retirement Study cohort to 0.046 in the LASA cohort. However, the associations between sex/gender and rate of frailty progression were mixed. There was significant heterogeneity in within-person trajectories of frailty about the mean curves.

**Discussion and Implications:**

Our findings of linear longitudinal increases in frailty highlight important avenues for future research. Specifically, we encourage further research to identify potential effect modifiers or groups that would benefit from targeted or personalized interventions.

**Translational Significance:** This article examines longitudinal trajectories of frailty and highlights the use of coordinated analytical methodology in multicohort studies. Understanding trajectories of frailty, and predisposing factors, is important to identify specific groups of individuals who would benefit from targeted or personalized interventions. In this study from five cohorts in the United States and Europe we observed linear trajectories of frailty. Model estimates in four of the five cohorts indicate that clinically meaningful increases in the frailty index may be observed within 5 years. Higher levels of frailty were observed in women compared to men, while education was associated with reduced levels of frailty.

## Background and Objectives

Frailty is defined as a state of increased vulnerability to adverse outcomes in older adults resulting from disorders of several physiological systems that eventually lead to the system being overwhelmed (Rockwood & Mitnitski, 2007). The prevalence of frailty in Europeans aged 50 and older is estimated to be around 18% (95% confidence interval [CI] 15%–21%). With current and forecasted increases in life expectancy combined with a modest change in health span, this figure will be even higher in the near future (O’Caoimh et al., 2018). A large body of literature indicates that frail individuals are at greater risk of adverse events such as falls, hospitalization, delirium, and mortality (Hoogendijk, Afilalo, et al., 2019). Hence, the need to improve our understanding of frailty and predisposing factors is critical. This knowledge will facilitate the design of effective interventions that may potentially prevent or delay the clinical consequences of frailty.

Numerous cross-sectional studies have identified various risk and protective factors for frailty. Still, a recent review (Welstead et al., 2020) reported a relatively limited understanding of longitudinal frailty progression and trajectories. Importantly, the review concluded that although trajectories of frailty tend to gradually worsen over time, the evidence regarding factors associated with these trajectories is mixed. Most notably, it found that evidence for three critical risk factors—age, sex, and, to a lesser extent, education—is inconsistent. For instance, in community-based studies, age is often found to be associated with frailty level and change, but the direction of the association varied by publication. While some reported that frailty progressed at a faster rate in older adults than in younger individuals (Peek et al., 2012; Rogers et al., 2017), others reported the opposite or did not find an association between older age and frailty progression (Hoogendijk et al., 2018; Mitnitski et al., 2012). Similarly, investigations of sex differences in frailty are scarce and inconsistent. For example, in a study of older Europeans who participated in the Survey of Health and Retirement in Europe, women accumulated health deficits faster than men (Stolz et al., 2017). Conversely, in an English sample of individuals of similar characteristics, women had higher frailty scores than men at each time point, but the rate of progression was not different (Marshall et al., 2015). A review by Gordon et al. (2017) indicates that women live for a longer period with higher levels of frailty. Indeed, women typically have higher levels of disability, comorbidities, and polypharmacy, and yet longer life expectancies than men (Corbi et al., 2019; Hubbard & Rockwood, 2011; Theou et al., 2014). This well-documented discrepancy of health and survival between men and women is termed the “male–female health survival paradox” (Gordon & Hubbard, 2018; Hubbard & Rockwood, 2011). Finally, the association between education and trajectories of frailty appears to be more consistent, although evidence is limited as the focus of only two studies. In both studies, a protective effect of education against frailty progression was reported (Chen et al., 2015; Peek et al., 2012).

The recent review identified various reasons that may explain inconsistencies in findings. In particular, they argued that discrepancies in the methodologies employed hamper the comparability of results across studies (Welstead et al., 2020). For instance, some studies quantified frailty using Fried et al.’s (2001) frailty phenotype, whereas other studies used the frailty index (FI). Consequently, results are not comparable: while the FI generates a continuous measure, the frailty phenotype classifies individuals into discrete states, and as a result, it is not possible to derive an estimate of a trajectory. Further, in studies where frailty trajectories were estimated, the use of different analytical techniques and adjustments for an inconsistent set of variables further hinder comparisons across findings.

A coordinated analytical approach has been proposed as a method to evaluate the consistency of findings across multiple studies and test for similarity of patterns of associations (Hofer & Piccinin, 2009). This approach involves the independent fit of the same analytical model and adjustment for the same set of variables with consistent coding to data from different cohorts. Furthermore, this approach generates independent results from each study data set with subsequent evidence synthesis of pooled estimates of interest and examination of study heterogeneity.

Here, we tested the hypothesis that there are no differences in frailty trajectories between men and women. With this purpose, we derived an FI in five longitudinal cohorts of older adults and, using a coordinated analytical approach, estimated trajectories of frailty in each of the cohorts, and examined the effects of age, sex/gender, and education on these trajectories.

## Research Design and Methods

### Data

Data were derived from five cohorts, the English Longitudinal Study of Aging (ELSA; Steptoe et al., 2013), the Health and Retirement Study (HRS; Juster & Suzman, 1995), the Invecchiare in Chianti (Aging in the Chianti area study; InCHIANTI; Ferrucci et al., 2000), the Longitudinal Aging Study Amsterdam (LASA; Hoogendijk, Deeg, et al., 2020), and the Rush Memory and Aging Project (MAP; Bennett et al., 2012, 2018). Participants were excluded from the analyses if they were aged under 65 or diagnosed with dementia at baseline. Each cohort was required to have at least three waves of data to allow us to model nonlinear trajectories (Singer et al., 2003), and to collate a wide range of health-related data such as cognitive function, activities of daily living, lifestyle, mental health, physical health, and motor function to allow calculation of an FI (Rockwood et al., 2005).

#### English Longitudinal Study of Aging

The ELSA is a representative sample of community-dwelling respondents aged 50 or older in England, UK. The ELSA sample was selected from participants of the Health Survey for England in 1998, 1999, and 2001. The ELSA baseline wave commenced in 2002, with biannual follow-up waves. All ELSA interviews were conducted face to face using computer-assisted interviewing, combined with self-completion questionnaires completed using pen and paper. The current study uses seven waves of data from 2002 to 2016 from the ELSA version E data set created using data from the 27th edition of ELSA, released March 2017. Participants were excluded if they did not participate in the 2002 measurement wave.

#### Health and Retirement Study

The HRS is a nationally representative longitudinal study of Americans over 50. The focus of the study is to provide data on the changing health and economic circumstances associated with aging. The baseline wave was in 1992 with follow-up waves every 2 years. Most baseline interviews were conducted face to face, with follow-ups primarily conducted over the telephone. This study uses only the primary respondents (no spouses) across all cohorts using the RAND HRS Longitudinal File 2016 (V2) from 10 waves between 1996 and 2016.

#### Invecchiare in Chianti

The InCHIANTI, aging in the Chianti area study is a prospective population-based cohort study among adults in Tuscany, Italy, with a large subsample aged 65 years and older. The study focuses on mobility decline and related factors in later life. The baseline wave commenced in 1998–2000, with follow-up waves every 3 years. Data are collected at each wave by a home interview and clinical measurements at the study clinic. The current study uses data from four waves between 1998–2000 and 2007–2009.

#### Longitudinal Aging Study Amsterdam

The LASA is a cohort study aimed at determining the predictors and consequences of physical, cognitive, emotional, and social functioning in older adults in the Netherlands. The LASA study consists of a nationally representative sample of older adults between 55 and 85. The data collection started in 1992–1993 with follow-up waves collected every 3 years. Data were collected at each wave by face-to-face interviews and clinical tests at the home of the participant. For the current study data were used from six waves between 1995–1996 and 2011–2012. Participants were excluded from the analysis if they did not participate in the 1995–1996 measurement wave.

#### Rush Memory and Aging Project

The MAP is a longitudinal community-based cohort study of older adults recruited from retirement and subsidized housing facilities, and individual homes in northeastern Illinois, United States. Participants consist of older adults without dementia who agree to annual clinical evaluations and organ donation at death. Recruitment began in 1997 and is ongoing. Participants are assessed annually by in-person assessments. Data from 20 waves between 1997 and 2017 were included in the current analyses.

### Frailty Index

To measure frailty we used the FI based on the accumulation of deficits approach (Rockwood & Mitnitski, 2007). Following the standard operating procedure defined by Searle et al. (2008), each FI requires a minimum of 30 deficits with each deficit fulfilling the following criteria: association with health status; present in each wave of data with no less than 5% missing data in each wave; prevalence should increase with age but should not saturate in the population before the age of 50; collective deficits should represent several different biological systems. Where possible, we used previously validated FI’s specific to each cohort (Hoogendijk et al., 2017, 2020; Mezuk et al., 2016; Warmoth et al., 2018); where this was not possible we created an FI using the above procedure of deficit selection. The deficits included in the FIs in this study were activities of daily living, cognition, comorbidities, mobility, self-reported health, instrumental activities of daily living, and physical health. All deficits were coded as 1 if present and 0 if absent. The total number of deficits was then divided by the number of items measured to produce an FI between 0 and 1, whereby higher values indicate higher levels of frailty. Participants were excluded if more than 20% of items comprising the FI were missing from the data. See Supplementary Tables 1–5 for a description of the FI for each cohort.

### Statistical Analysis

We estimated five independent latent growth curve models to repeated measures of the FI in each of the five cohorts. In these models, the intercept and change parameters were adjusted for age, sex/gender, and years of education. We include the term sex/gender as a covariate to model the combined association of biological or social mechanisms, both of which may contribute to frailty trajectories in women. Importantly, data collection regarding sex and gender in the five cohorts is unclear, making the distinction between biological sex and gender unfeasible and further necessitating the use of the combined association; this would not affect findings from the models used. For data harmonization, sex/gender was recoded as 0 for male and 1 for females in each cohort.

We first estimated models for age-related linear trajectories of change in all cohorts. The model intercept was placed at the age of 65 years. Intercept and slope were adjusted for age (centered at 65), sex/gender (F = 1, M = 0), and years of education (centered at 7 years). With this parameterization, the intercept of the linear model represents the level of frailty of a 65-year-old man with 7 years of education who entered the study at age 65, and the slope represents the annual rate of frailty change. Next, we included a sex/gender-by-age interaction term to the first linear model to gain insights into the male–female health paradox.

Third, we estimated models describing a quadratic trajectory of frailty. In this case, the interpretation of the intercept remains unchanged. However, the linear slope is now interpreted as the rate of change at the age of 65 years of age (intercept), and the quadratic slope as the rate of change in the linear slope over the study follow-up time. The fourth model added a sex/gender-by-age interaction term to the quadratic model.

Once all four models were estimated, model selection was performed comparing Bayesian Information Criteria (BIC; Schwarz, 1978) values obtained from each model. The BIC is a tool for selecting the most parsimonious, best-fitting model based on a combination of the model likelihood penalized by the number of parameters estimated. The model with the lowest BIC is preferred (Raftery, 1995). All models were estimated using maximum likelihood estimation under a missing at random missing data assumption, using MPLUS version 8.1 (B. O. Muthén, 2018; L. K. Muthén & Muthén, 1998). Finally, after selecting the models with the best fit for each cohort, we considered a nonlinear effect of baseline age by including an age-squared term to the final best-fitting models.

## Results

### Descriptive Characteristics Across Cohorts

Cohort characteristics are presented in Table 1. There were differences across the studies in age at baseline, levels of the FI, and years of education. For instance, an analysis of variance showed statistically significant differences of age at baseline across cohorts (*F*_(4)_ = 257.01, *p* < .001). Further, the mean age at baseline ranged from 74 in the ELSA cohort to 80 in the MAP cohort. Similar analyses showed differences in the mean FI across cohorts at baseline (*F*_(4)_ = 45.05, *p* < .001); the mean FI at baseline ranged from 0.13 in the MAP cohort to 0.20 in the LASA cohort. Finally, differences were also observed in years of formal education (*F*_(4)_ = 2,255.88, *p* < .001), which ranged from 5 in the ELSA cohort to 15 in the MAP cohort.

**Table 1.** Descriptive Characteristics of the Cohorts at Baseline

Cohort name	Country	FI	Total sample (*N*)	No. waves	Data collection period	Follow-up cycle	Mean age (*SD*)	Mean years of education (*SD*)	% Male	Mean FI (*SD*)
ELSA	United Kingdom	38-item	5,097	7	2002–2016	2 years	73.99 (6.57)	4.86 (6.57)	45.40	0.16 (0.15)
HRS	United States	30-item	8,234	10	1996–2016	2 years	76.41 (7.06)	11.04 (3.71)	42.70	0.19 (0.26)
InCHIANTI	Italy	42-item	1,132	4	1998–2009	3 years	75.19 (7.44)	5.33 (3.32)	43.20	0.17 (0.13)
LASA	The Netherlands	32-item	1,742	6	1995–2012	3 years	76.00 (6.69)	8.77 (3.31)	46.40	0.20 (0.12)
MAP	United States	41-item	1,738	20	1997–2017	Annual	79.96 (7.60)	14.68 (3.29)	26.30	0.13 (0.16)

*Notes*: ELSA = English Longitudinal Cohort Study; FI = frailty index; HRS = Health and Retirement Study; InCHIANTI = Invecchiare in Chianti Study; LASA = Longitudinal Aging Study Amsterdam; MAP = Rush Memory and Aging Project; *SD* = standard deviation.

### Differences in Frailty Trajectories Across Cohorts

**Figure 1.**
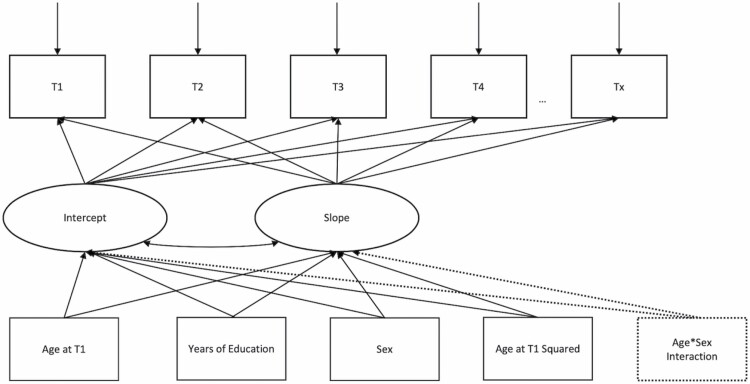
A path diagram representing the final models for each cohort. The dotted lines represent the additional adjustment for an age–sex/gender interaction in the final model for the Rush Memory and Aging Project (MAP) cohort only.

Supplementary Table 7 displays the BIC indices for each of the four models estimated for each cohort. According to the BIC, in all cohorts, models describing linear progression of frailty were preferred. For MAP, the linear model adjusting for an age–sex/gender interaction was the best-fitting model; for all other cohorts, the linear model without an age–sex/gender interaction was the best-fitting model. The best-fitting model for each cohort was then adjusted for age-squared, representing the final models. The final models are represented in Figure 1.

The HRS cohort was found to have the highest level of frailty at 65 years of age of all studies included here. On average, the FI for a reference individual in the HRS cohort (a 65-year-old male, with 7 years of education) was estimated at β = 0.171 (*SE* = 0.007), with an annual rate of increase of β = 0.008 (*SE* = 0.001). By contrast, the lowest level of frailty for the reference person was observed in the ELSA cohort (β = 0.088 [*SE* = 0.006], *p* < .001), with an annual rate of deficit increase of 0.006 (*SE* = 0.000).

The fastest rate of change in FI was observed in the LASA cohort (β = 0.009 [*SE* = 0.001], *p* < .001), and the InCHIANTI cohort had the slowest rate of change in FI (β = 0.002 [*SE* = 0.001]). Results from the linear models are presented in Table 2.

**Table 2.** Results From the Final Growth Curve Models Representing the Trajectories of the Frailty Index

Variable	ELSA		HRS		InCHIANTI		LASA		MAP	
	Linear		Linear		Linear		Linear		Linear (with sex/gender × age interaction)	
	β	SE	β	SE	β	SE	β	SE	β	SE
Fixed effects										
Intercept	0.088***	0.006	0.171***	0.007	0.089***	0.007	0.126***	0.010	0.112***	0.018
Sex/gender	0.035***	0.006	0.014*	0.005	0.018*	0.007	0.046***	0.008	0.022	0.021
Education	−0.003***	+0.000	−0.008***	0.001	−0.002	0.001	−0.003*	0.001	−0.004*	0.002
Baseline age	0.004	0.002	−0.008***	0.001	+0.000	0.002	−0.008*	0.003	−0.004	0.003
Baseline age^2^	−0.001***	+0.000	−0.001***	+0.000	−0.001***	+0.000	+0.000	+0.000	−0.001***	+0.000
Age × sex/gender	n/a	n/a	n/a	n/a	n/a	n/a	n/a	n/a	−0.002	0.002
Linear growth rate	0.006***	+0.000	0.008***	0.001	0.002*	0.001	0.009***	0.001	0.005*	0.001
Sex/gender	+0.000	+0.000	0.001*	+0.000	+0.000	0.001	−0.001	0.001	−0.001	0.002
Education	+0.000*	+0.000	+0.000*	+0.000	0.000*	+0.000	+0.000	+0.000	+0.000	+0.000
Baseline age	+0.000***	+0.000	0.001***	+0.000	0.001***	+0.000	0.001***	+0.000	0.001***	+0.000
Baseline age^2^	+0.000***	+0.000	+0.000	+0.000	+0.000	+0.000	+0.000	+0.000	+0.000	+0.000
Age × sex/gender	n/a	n/a	n/a	n/a	n/a	n/a	n/a	n/a	+0.000	+0.000
Random effects										
Intercept	0.019***	0.001	0.027***	0.001	0.001	0.001	0.011***	0.001	0.014***	0.002
Linear growth rate	+0.000***	+0.000	+0.000***	+0.000	+0.000***	+0.000	+0.000***	+0.000	+0.000***	+0.000
Residual	0.006***	+0.000	0.009***	+0.000	0.004***	+0.000	0.003***	+0.000	0.005***	+0.000
Goodness of fit (BIC):	−34154.843		−51844.470		−6243.470		−10985.030		−23009.679	

*Notes*: β = coefficient; BIC = Bayesian Information Criterion; ELSA = English Longitudinal Cohort Study; HRS = Health and Retirement Study; InCHIANTI = Invecchiare in Chianti Study; LASA = Longitudinal Aging Study Amsterdam; MAP = Rush Memory and Aging Project; *SE* = standard error. Mplus software uses double precision, using seven digits in calculations. As such, even when values are 0.000, the software can still determine significance. In these cases, directionality is indicated by + or −.

*p < .05. ***p < .001.

### Factors Associated With FI Level and Rate of Change

**Figure 2.**
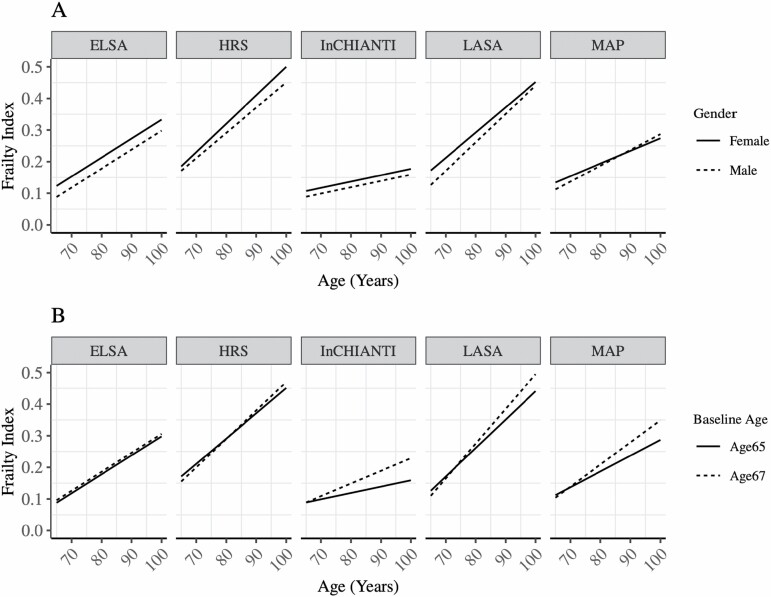
Graphical representation of the estimated model trajectory of the frailty index across cohorts for: (A) female and male; (B) individuals aged 65 or 67 at baseline.

Across all five cohorts, women were found to have higher levels of frailty than men; this was significant in all cohorts except MAP. The effect size for sex/gender differences ranged from β = 0.046 (*SE* = 0.008) in the LASA cohort to β = 0.014 (*SE* = 0.005) in the HRS cohort. However, sex/gender differences on annual frailty progression were not consistent in direction across cohorts. No sex/gender differences in rate of frailty progression emerged in the LASA, MAP, InCHIANTI, or ELSA cohorts. However, in the HRS cohort, the annual increase of frailty in women was faster than in men. Figure 2A compares model trajectories of FI for male and female participants across all five cohorts.

Older age at baseline revealed mixed effects in frailty levels across the cohorts. In the HRS, LASA, and MAP cohorts each additional year of age at baseline was associated with lower levels of frailty. In the ELSA and InCHIANTI cohorts each additional year of age at baseline was not associated with levels of frailty. The association between baseline age and rate of change in frailty was statistically significant in all cohorts, although negligible in ELSA, the youngest cohort. Figure 2B shows the effect of an additional 2 years of age at baseline compared to a reference individual aged 65 across each of the five cohorts. Furthermore, our results identify a nonlinear effect of age at study entry on frailty levels in all cohorts except for LASA and on the rate of frailty change only in ELSA.

Finally, education was associated with reduced frailty levels in all cohorts except InCHIANTI. The effect of education on the rate of change while minimal was significant in the ELSA, HRS, and InCHIANTI cohorts (see Table 2). In these cohorts each additional year of education was associated with small increases in the rate of deficit accumulation.

### Random Effects

In all cohorts except InCHIANTI, random effects about the intercept and rate of change were significant, suggesting that in all samples, heterogeneity in individual trajectories about mean curves exists. In the InCHIANTI cohort, while the random effect about the rate of change in FI was significant, the random effects about the intercept were not, suggesting that at age 65, there is homogeneity in individual levels of frailty but heterogeneity in individual trajectories of FI.

## Discussion and Implications

We estimated frailty trajectories in five large longitudinal studies of older adults and tested the hypothesis that there were no sex/gender differences in the rate of frailty progression. Our findings partially support our hypothesis regarding sex differences in frailty trajectories. Although our results show that in all five studies, women consistently have higher levels of frailty at age 65 years, results regarding sex/gender differences in the rate of frailty progression were mostly null. Only in HRS did women have faster rates of frailty progression than men.

Our findings regarding sex/gender differences in frailty levels agree with existing evidence that women reach older age with more deficits than men (Mitnitski et al., 2005; Stolz et al., 2017; Theou et al., 2014). Indeed, a systematic review and meta-analysis of seven population-based studies reported consistently higher FI scores in women than in men (Gordon et al., 2017). Previous literature also found that women outlive men despite having higher levels of frailty at age 65 years as well as higher levels of disability, comorbidities, and polypharmacy (Corbi et al., 2019; Hubbard & Rockwood, 2011; Theou et al., 2014). This discrepancy between health and survival in men and women is known as the ‘male–female health survival paradox’ (Gordon et al., 2017; Hubbard & Rockwood, 2011). It has been partially explained by physiological differences between men and women (Gordon & Hubbard, 2018). Our results concerning the association of sex/gender (as well as age and education) with the rate of frailty progression were inconsistent between cohorts. This is also the case in previous literature: while Stolz et al. (2017) observed a faster accumulation of deficits in females compared to males in the Survey Health Aging and Retirement in Europe cohort, Marshall et al. (2015), using the ELSA cohort, did not. Importantly, these results need to be interpreted with caution as estimates were minimal, and most were nonsignificant. This is likely a consequence of the scale of the FI (which yields values between 0 and 1) and the slow accumulation of deficits.

Education was consistently found to be associated with lower levels of frailty at study entry. In three of the five studies, it was also associated with a slower rate of frailty progression, although the estimates were minimal. Previous research on the association between education and longitudinal frailty trajectories is relatively limited, but the two existing reports identified in the review by Welstead et al. (2020) agree with our findings (Chen et al., 2015; Peek et al., 2012). It is likely that more educated individuals engage in healthier lifestyles in midlife and over the life course, and therefore, reach older ages with fewer deficits (Gil-Salcedo et al., 2020).

In four of the five cohorts, we found a nonlinear association between frailty levels and baseline age, with negative estimates of the quadratic terms in most cases, suggesting that baseline frailty differences due to age at baseline become smaller at the extremes of the age distribution. While this seems counterintuitive, the healthy participant effect (Sedgwick, 2012) may explain these results. According to the healthy participant effect, individuals who join studies at an older age tend to be healthier than younger study participants. Equally, frail individuals have high mortality and higher levels of attrition, and those with faster rates of progression are likely removed from the population.

Clinically meaningful change in frailty indices have been estimated to range between 0.02 and 0.076, whereby 0.02–0.03 represents a small clinically meaningful change, and 0.049–0.076 represents large clinically meaningful change (Jang et al., 2020). Across all five studies, frailty progressed linearly. While the average rate of change in FI may appear relatively small, our results suggest that within 5 years small clinically meaningful increases in FI in ELSA and MAP, and large clinically meaningful increases in FI in LASA and HRS may occur. However, the concept of a clinically meaningful change needs to be considered with caution. There is no consensus on a single definition for clinically meaningful differences, and the degree of meaningful change will vary depending on various factors such as baseline level, age, sex, etc. (Keefe et al., 2013). Interestingly, LASA had the fastest rate of increase and the highest level of frailty at baseline. Various reasons may explain these results. For instance, the deficits included in the derivation of the FI in LASA may be more prevalent in the population than in the other samples. In addition, it is possible that individuals with higher frailty levels at baseline die or dropout after the initial wave and those who remain in the study have not yet reached saturation of deficits, and hence, accumulate deficits faster than in other studies. Although previous reports have suggested that the FI is robust to differences in the index composition, the comparison of results across samples where indices are derived with different deficits has not been explored extensively in the literature (Shi et al., 2020) and is an area that merits further future exploration.

Our work has some limitations. The analyses performed assume missing data are missing at random, which may not be a realistic assumption in studies of older adults (though is almost universally made). We also only included a basic set of variables in the models to maximize comparability of results, losing the ability to further exploit the richness of data available in each cohort. This was done to maximize opportunities to assess the consistency of results across studies. Nevertheless, some methodological aspects still need consideration. First, the studies included in our analyses had long follow-up periods that ranged from 11 to 20 years. During such prolonged periods, frailty progression may reach a steady state, and hence, the best-fitting curve is a linear one. On the contrary, it is possible that the linearity in the process may be a consequence of how the index is derived. That is, as the index does not leverage the relative weight of the domains included in this derivation, which may be correlated, it is possible that an initial saturation of deficits occurs in a heavily represented domain. As a result, fewer deficits remain to be accumulated, and therefore contribute, to the later progression of frailty, which would result in quasilinear increases. Given the current limited understanding of the ordering of deterioration across systems, the disentanglement of this conundrum is a pending task. New methodologies, such as network analyses, offer meaningful opportunities to further existing knowledge about frailty and the possible impact of correlation between deficits within and across domains in frailty progression (Rutenberg et al., 2018). Finally, in all five cohorts, data collection regarding sex/gender is unclear. The exact phrasing of the question, if indeed asked, is not available, making the distinction between biological sex and gender unfeasible. This necessitated our approach to combine the associations of sex and gender. Arguably, in the context of frailty both biological sex and gender are relevant, and may influence prevalence and disease progression; however, they are not interchangeable. In concluded legacy cohorts, this will remain an issue; however, ongoing cohorts should address this important issue in subsequent waves.

Our findings of linear increases in frailty across five longitudinal studies of aging with prolonged follow-up highlight important avenues for future research. Specifically, we encourage further research to fully comprehend the impact of domain-specific contributions on different frailty progression between men and women to improve the design of effective interventions to delay frailty in older adults. For instance, given the documented differences in engagement of healthy behaviors in men and women (Baumann et al., 2017; Ek, 2015), a better understanding of how they may differently relate to frailty progression in men and women would support tailored earlier life interventions to slow down frailty progression.

Further research is needed to identify potential effect modifiers or groups that would benefit from targeted or personalized interventions. By enhancing our understanding of frailty trajectories our findings have the potential to inform the design of interventions to reduce frailty-related adverse events such as falls, hospitalization, and mortality.

## Supplementary Material

igab059_suppl_Supplementary_Material
