# Supplementary material for: Trajectories of Frailty With Aging: Coordinated Analysis of Five Longitudinal Studies
Source: Innov Aging. 2022 Jan 15;6(2):igab059. doi: 10.1093/geroni/igab059 (PMC8882228; doi:10.1093/geroni/igab059)
Supplement: igab059_suppl_Supplementary_Material [file igab059_suppl_supplementary_material.docx]

***Innovation in Aging* Supplementary Material:** Trajectories of frailty with aging: Coordinated analysis of five longitudinal studies

[**Table 1.** Composition of the ELSA Frailty Index 2](#_Toc85393088)

[**Table 2.** Composition of the HRS Frailty Index 3](#_Toc85393089)

[**Table 3.** Composition of the InCHIANTI Frailty Index 4](#_Toc85393090)

[**Table 4.** Composition of the LASA Frailty Index 5](#_Toc85393091)

[**Table 5.** Composition of the MAP Frailty Index 6](#_Toc85393092)

[**Table 6.** Cohort sample sizes across waves of data collection 7](#_Toc85393093)

[**Table 7.** BIC Indices for Quadratic and Linear FI trajectory models 7](#_Toc85393094)

**Table 1.** Composition of the ELSA Frailty Index

|  | **Deficit** | **Cut-off** | |  |
| --- | --- | --- | --- | --- |
| **ADLs** |  |  | |  |
| 1 | Difficulty dressing | No=0, yes=1 | |  |
| 2 | Difficulty walking across a room | No=0, yes=1 | |  |
| 3 | Difficulty bathing or showering | No=0, yes=1 | |  |
| 4 | Difficulty getting in and out of bed | No=0, yes=1 | |  |
| 5 | Difficulty using the toilet | No=0, yes=1 | |  |
| 6 | Difficulty eating | No=0, yes=1 | |  |
| **Comorbidities** | | |  | |
| 7 | High blood pressure/ Hypertension | No=0, yes=1 | |  |
| 8 | Heart problems (including angina) | No=0, yes=1 | |  |
| 9 | Diabetes or high blood sugar | No=0, yes=1 | |  |
| 10 | Stroke history | No=0, yes=1 | |  |
| 11 | Lung disease | No=0, yes=1 | |  |
| 12 | Asthma | No=0, yes=1 | |  |
| 13 | Arthritis/ Osteoporosis | No=0, yes=1 | |  |
| 14 | Cancer | No=0, yes=1 | |  |
| 15 | Psychiatric disease | No=0, yes=1 | |  |
| 16 | Cataracts | No=0, yes=1 | |  |
| **IADLs** |  |  | |  |
| 17 | Difficulty making telephone calls | No=0, yes=1 | |  |
| 18 | Difficulty taking medications | No=0, yes=1 | |  |
| 19 | Difficulty using the map to figure out to get around | No=0, yes=1 | |  |
| 20 | Difficulty preparing a hot meal | No=0, yes=1 | |  |
| 21 | Difficulty shopping for groceries | No=0, yes=1 | |  |
| 22 | Difficulty managing money | No=0, yes=1 | |  |
| 23 | Difficulty doing work around the house and garden | No=0, yes=1 | |  |
| **Mobility** | | | |  |
| 24 | Difficulty walking 100 yards | No=0, yes=1 | |  |
| 25 | Difficulty sitting for 2 hours | No=0, yes=1 | |  |
| 26 | Difficulty getting up from a chair after sitting | No=0, yes=1 | |  |
| 27 | Difficulty climbing several flights of stairs | No=0, yes=1 | |  |
| 28 | Difficulty climbing one flight of stairs | No=0, yes=1 | |  |
| 29 | Difficulty stooping, kneeling or crouching | No=0, yes=1 | |  |
| 30 | Difficulty reaching or extending arms above shoulders | No=0, yes=1 | |  |
| 31 | Difficulty pulling or pushing large objects | No=0, yes=1 | |  |
| 32 | Difficulty picking up 5p coin from table | No=0, yes=1 | |  |
| 33 | Difficulty lifting/ carrying 10lbs | No=0, yes=1 | |  |
| **Self-reported health** | | |  | |
| 34 | Self-reported general health | Excellent-fair = 0, poor = 1 | |  |
| 35 | Felt depressed in last week | No=0, yes=1 | |  |
| 36 | Sleep restless in last week | No=0, yes=1 | |  |
| 37 | Self-reported poor eyesight | No=0, yes=1 | |  |
| 38 | Self-reported poor hearing | No=0, yes=1 | |  |

a. ADLs, Activities of daily living; ELSA, English Longitudinal Cohort Study; FI, Frailty Index; IADLs, Instrumental activities of daily living.

b. The items in the ELSA FI were adapted from the FI derived by Warmoth et al [2018] in the ELSA dataset. Items related to cognition (diagnosis of Alzheimer’s disease, diagnosis of dementia, and cognitive functioning test items) were removed, and three additional items were included: cataracts, depression symptoms and sleep problems. This resulted in a frailty index comprised of 38 deficits.

**Table 2.** Composition of the HRS Frailty Index

|  | **Deficit** | **Cut-off** |
| --- | --- | --- |
| **ADLs** | |  |
| 1 | Difficulty getting dressed | No = 0, Yes = 1 |
| 2 | Difficulty with bathing/showering | No = 0, Yes = 1 |
| 3 | Difficulty using toilet | No = 0, Yes = 1 |
| 4 | Difficulty preparing hot meals | No = 0, Yes = 1 |
| 5 | Difficulty shopping for groceries | No = 0, Yes = 1 |
| 6 | Change in activities of daily living | No = 0, Yes = 1 |
| 7 | Incontinence (past 12 months) |  |
| **Comorbidities** | |  |
| 8 | Hypertension | No = 0, Yes = 1 |
| 9 | Myocardial infarction | No = 0, Yes = 1 |
| 10 | Congestive heart failure | No = 0, Yes = 1 |
| 11 | Other cardiac problems | No = 0, Yes = 1 |
| 12 | History of stroke | No = 0, Yes = 1 |
| 13 | History of diabetes mellitus | No = 0, Yes = 1 |
| 14 | Long-term memory impairment | No = 0, Yes = 1 |
| 15 | Arthritis | No = 0, Yes = 1 |
| 16 | Cancer | No = 0, Yes = 1 |
| 17 | Respiratory problems | No = 0, Yes = 1 |
| 18 | History of major psychiatric disorder | No = 0, Yes = 1 |
| 19 | Lung disease | No = 0, Yes = 1 |
| **Mobility** | |  |
| 20 | Difficulty walking across room, walking several blocks, climbing stairs | No = 0, Yes = 1 |
| 21 | Falls (past 2 years) | No = 0, Yes = 1 |
| 22 | Difficulty in large muscle activities (e.g., stooping, chair stand, kneeling, pushing large object) | No = 0, Yes = 1 |
| 23 | Difficulty in fine motor skills such as picking up a dime, eating and dressing | No = 0, Yes = 1 |
| 24 | Back pain or back problems | No = 0, Yes = 1 |
| **Self-reported health** | |  |
| 25 | Memory worse than it was 2 years ago | No = 0, Yes = 1 |
| 26 | Persistent Headache | No = 0, Yes = 1 |
| 27 | Trouble sleeping | No = 0, Yes = 1 |
| 28 | Tiredness all the time | No = 0, Yes = 1 |
| 29 | Feeling sad, blue, depressed | No = 0, Yes = 1 |
| 30 | Trouble with pain | No = 0, Yes = 1 |

a. ADLs, Activities of daily living; FI, Frailty Index; HRS, Health and Retirement Study*.*

b. The HRS FI is a validated FI, consisting of 30 items that were available across the waves of the “RAND HRS Longitudinal File 2016 (V2)” [Mezuk et al., 2017].

**Table 3.** Composition of the InCHIANTI Frailty Index

|  | **Deficit** | **Cut-Off** |
| --- | --- | --- |
| **ADLs** | |  |
| 1 | Bathing | No difficulty = 0, with difficulty but without help = 0.33 with some help from another person = 0.66 unable to do it=1 |
| 2 | Dressing/undressing |  |
| 3 | Eating |  |
| 4 | Toileting |  |
| 5 | Continence |  |
| 6 | Walking across small room |  |
| 7 | Walking up/down staircase ten steps |  |
| 8 | Getting in/out of bed |  |
| **Cognition** | |  |
| 9 | Orientation time (MMSE) | Five correct = 0, one wrong = 0.50, two or more wrong = 1 |
| 10 | Orientation place (MMSE) | Five correct = 0, one wrong = 0.50, two or more wrong = 1 |
| 11 | Attention (MMSE) | Five correct = 0, one or two wrong = 0.50, three or more wrong = 1 |
| 12 | Recall (MMSE) | Three correct = 0, two correct = 0.50, one or zero correct = 1 |
| **Comorbidities** | |  |
| 13 | Hypertension | No=0, possible=0.5, yes=1 |
| 14 | Myocardial infarction | No=0, possible=0.5, yes=1 |
| 15 | Congestive heart failure | No=0, possible=0.5, yes=1 |
| 16 | Chronic liver disease | No=0, possible=0.5, yes=1 |
| 17 | Cancer | No=0, yes=1 |
| 18 | Peripheral arterial disease | No=0, possible=0.5, yes=1 |
| 19 | Stroke | No = 0, possible/TIA = 0.5, yes = 1 |
| 20 | Parkinson`s disease | No=0, possible=0.5, yes=1 |
| 21 | Diabetes | No=0, possible=0.5, yes=1 |
| 22 | Chronic lung disease | No=0, possible=0.5, yes=1 |
| 23 | Angina pectoris | No=0, possible=0.5, yes=1 |
| 24 | Knee/hip arthritis | No = 0, pain or stiffness = 0.5, pain and stiffness = 1 |
| **IADLs** | |  |
| 25 | Food preparation | No difficulty = 0, with difficulty but without help = 0.33 with some help from another person = 0.66 unable to do it=1 |
| 26 | Shopping |  |
| 27 | Heavy housework |  |
| 28 | Using telephone |  |
| 29 | Lifting/carry shopping bag |  |
| 30 | Using public transportation |  |
| 31 | Medication use |  |
| 32 | Managing finances |  |
| **Physical Health** | |  |
| 33 | Weight loss | No = 0, yes, (weight loss > 10 lbs. in past year) = 1 |
| 34 | Low physical activity | No = 0, yes (hardly any physical activity or < 1 h a week) = 1 |
| 35 | Slow gait speed | Normal = 0, lowest quintile, stratified by height and sex = 1 |
| 36 | Low grip strength | Normal = 0, lowest quintile, stratified by BMI and sex = 1 |
| **Self-reported health** | |  |
| 37 | Self-rated health | Very good = 0, good = 0.25, fair = 0.50, poor = 0.75, very poor = 1 |
| 38 | Feel depressed (CES-D) | Rarely or never = 0, sometimes = 0.33, occasionally = 0.66, often or always = 1 |
| 39 | Feel everything is an effort (CES-D) | Rarely or never = 0, sometimes = 0.33, occasionally = 0.66, often or always = 1 |
| 40 | Could not get going (CES-D) | Rarely or never = 0, sometimes = 0.33, occasionally = 0.66, often or always = 1 |
| 41 | Feel lonely (CES-D) | Rarely or never = 0, sometimes = 0.33, occasionally = 0.66, often or always = 1 |
| 42 | Feel happy (CES-D) | Often or always = 0, occasionally = 0.33, sometimes = 0.66, rarely or never = 1 |

a. ADLs, Activities of daily living; CES-D, Center for Epidemiologic Studies Depression Scale; FI, Frailty Index; IADLs, Instrumental activities of daily living; InCHIANTI, Invecchiare in Chianti Study; MMSE, Mini-Mental State Examination.

b. The InCHIANTI FI used in these analyses is a previously validated 42-item frailty index from the InCHAINTI cohort [Hoogendijk et al., 2020].

**Table 4.** Composition of the LASA Frailty Index

|  | **Deficit** | **Cut-Off** |
| --- | --- | --- |
| **ADLs** | |  |
| 1 | Dress/undress self | Yes= 0, some difficulty= 0.25, much difficulty= 0.50, only with help= 0.75, no= 1 |
| 2 | Sit down/stand up from chair | Yes= 0, some difficulty= 0.25, much difficulty= 0.50, only with help= 0.75, no= 1 |
| 3 | Cut own toenails | Yes= 0, some difficulty= 0.25, much difficulty= 0.50, only with help= 0.75, no= 1 |
| **Cognition** | |  |
| 4 | Memory complaints | No = 0, yes = 1 |
| 5 | Orientation time (MMSE) | Five correct = 0, one wrong = 0.50, two or more wrong = 1 |
| 6 | Orientation place (MMSE) | Five correct = 0, one wrong = 0.50, two or more wrong = 1 |
| 7 | Attention (MMSE) | Five correct = 0, one or two wrong = 0.50, three or more wrong = 1 |
| 8 | Recall (MMSE) | Three correct = 0, two correct = 0.50, one or zero correct = 1 |
| **Comorbidities** | |  |
| 9 | Cardiac disease | No=0, yes=1 |
| 10 | Peripheral arterial disease | No=0, yes=1 |
| 11 | Stroke | No=0, yes=1 |
| 12 | Diabetes | No=0, yes=1 |
| 13 | Lung disease | No=0, yes=1 |
| 14 | Cancer | No=0, yes=1 |
| 15 | Arthritis | No=0, yes=1 |
| 16 | Hypertension | No=0, yes=1 |
| 17 | Other chronic disease 1 | No=0, yes=1 |
| 18 | Other chronic disease 2 | No=0, yes=1 |
| 19 | Incontinence | No=0, yes=1 |
| **IADLs** | |  |
| 20 | Use of transportation | Yes= 0, some difficulty= 0.25, much difficulty= 0.50, only with help= 0.75, no= 1 |
| **Mobility** | |  |
| 21 | Walk outside 5 min without stopping | Yes= 0, some difficulty= 0.25, much difficulty= 0.50, only with help= 0.75, no= 1 |
| 22 | Walk up/down staircase 15 steps without resting | Yes= 0, some difficulty= 0.25, much difficulty= 0.50, only with help= 0.75, no= 1 |
| **Physical Health** | |  |
| 23 | Physical activity (LAPAQ) | High (5+ activities) = 0, medium (3–4) = 0.33, low (1–2) = 0.66, no activities = 1 |
| 24 | Gait speed (6 m) | Normal = 0, slow (10 s) or physical unable = 1 |
| **Self-reported health** | |  |
| 25 | How is your health in general? | Excellent= 0, good= 0.25, fair= 0.50, sometimes good/bad= 0.75, Poor= 1 |
| 26 | How is your health compared to other people of your age? | Much /a little better= 0, just as good= 0.33, a little worse= 0.66, much worse = 1 |
| 27 | Feel depressed (CES-D) | Rarely/never= 0, some of the time= 0.33, occasionally= 0.66, mostly/always = 1 |
| 28 | Feel everything is an effort (CES-D) | Rarely/never= 0, some of the time= 0.33, occasionally= 0.66, mostly/always = 1 |
| 29 | Feel happy (CES-D) | Rarely/never= 0, some of the time= 0.33, occasionally= 0.66, mostly/always = 1 |
| 30 | Feel lonely (CES-D) | Rarely/never= 0, some of the time= 0.33, occasionally= 0.66, mostly/always = 1 |
| 31 | Enjoy life (CES-D) | Rarely/never= 0, some of the time= 0.33, occasionally= 0.66, mostly/always = 1 |
| 32 | Could not get going (CES-D) | Rarely/never= 0, some of the time= 0.33, occasionally= 0.66, mostly/always = 1 |

a. ADLs, Activities of daily living; CES-D, Center for Epidemiologic Studies Depression Scale; FI, Frailty Index; IADLs, Instrumental activities of daily living; LAPAQ, LASA Physical Activity Questionnaire; LASA, Longitudinal Aging Study Amsterdam; MMSE, Mini-Mental State Examination.

b. The LASA FI used in these analyses was a previously validated 32-item frailty index from the LASA cohort [Hoogendijk et al., 2017].

**Table 5.** Composition of the MAP Frailty Index

|  | **Deficit** | **Cut-off** |
| --- | --- | --- |
| **ADLs** | |  |
| 1 | Walking across small room | Does not need help = 0, needs help = 1 |
| 2 | Bathing | Does not need help = 0, needs help = 1 |
| 3 | Dressing | Does not need help = 0, needs help = 1 |
| 4 | Eating | Does not need help = 0, needs help = 1 |
| 5 | Getting up from bed | Does not need help = 0, needs help = 1 |
| 6 | Toilet | Does not need help = 0, needs help = 1 |
| **Comorbidities** | |  |
| 7 | Hypertension | No = 0, yes =1 |
| 8 | Diabetes | No = 0, yes =1 |
| 9 | Congestive heart failure | No = 0, yes =1 |
| 10 | Heart conditions | No = 0, yes =1 |
| 11 | Stroke | No = 0, yes =1 |
| 12 | Cancer | No = 0, yes =1 |
| 13 | Claudication (pain in legs) | No = 0, yes =1 |
| **IADLs** | |  |
| 14 | Using telephone | Does not need help = 0, needs help = 1 |
| 15 | Preparing meals | Does not need help = 0, needs help = 2 |
| 16 | Light housekeeping | Does not need help = 0, needs help = 3 |
| 17 | Heavy housekeeping | Does not need help = 0, needs help = 4 |
| 18 | Taking medication | Does not need help = 0, needs help = 5 |
| 19 | Finances | Does not need help = 0, needs help = 6 |
| 20 | Travel within community | Does not need help = 0, needs help = 7 |
| 21 | Shopping | Does not need help = 0, needs help = 8 |
| **Mobility** | |  |
| 22 | Walk up and down stairs (Rosow-Breslau Scale) | Does not need help = 0, needs help = 1 |
| 23 | Walk half a mile (Rosow-Breslau Scale) | Does not need help = 0, needs help = 1 |
| 24 | Heavy housework (Rosow-Breslau Scale) | Does not need help = 0, needs help = 1 |
| **Physical Health** | |  |
| 25 | Grip strength | Lowest quintile = 1 |
| 26 | Gait speed (8ft, 2.4m) | Lowest quintile = 1 |
| 27 | Pulmonary function (FEV/FVC) | Greater than .70 = 0, .70 or less = 1 |
| 28 | Vision | Visual acuity of 20/100 or better = 0, 20/200 or worse = 1 |
| 29 | Olfaction | Normal (11-12) = 0, Hyposmic (6-10) = .5, Anosmic (0-5) = 1 |
| 30 | Low BMI | BMI 20+ = 0, BMI < 20 = 1 |
| **Self-reported health** | |  |
| 31 | Felt depressed (CES-D) | No = 0, yes =1 |
| 32 | Everything was an effort (CES-D) | No = 0, yes =1 |
| 33 | Restless sleep (CES-D) | No = 0, yes =1 |
| 34 | Felt lonely (CES-D) | No = 0, yes =1 |
| 35 | People were unfriendly (CES-D) | No = 0, yes =1 |
| 36 | Felt sad (CES-D) | No = 0, yes =1 |
| 37 | Felt that others disliked me (CES-D) | No = 0, yes =1 |
| 38 | Could not get going (CES-D) | No = 0, yes =1 |
| 39 | Felt happy (CES-D) | No = 1, yes = 0 (Reverse coded CES-D item) |
| 40 | Enjoyed life (CES-D) | No = 1, yes = 0 (Reverse coded CES-D item) |
| 41 | Clinical depression | Not present = 0, possible = .33, probable = .66, highly probable = 1 |

a. ADLs, Activities of daily living; BMI, Body Mass Index; CES-D, Center for Epidemiologic Studies Depression Scale; FEV, Forced Expiratory Volume; FI, Frailty Index; FVC, Forced Vital Capacity; IADLs, Instrumental Activities of Daily Living; MAP, Rush Memory and Aging Project.

b. No previously validated FI was available for the MAP cohort, as such this 41-item FI was created following the procedure outlined by Searle et al (2008).

**Table 6.** Cohort sample sizes across waves of data collection

| **Cohort** | **Sample Size** | | | | | | | | | | | | | | | | | | | | |
| --- | --- | --- | --- | --- | --- | --- | --- | --- | --- | --- | --- | --- | --- | --- | --- | --- | --- | --- | --- | --- | --- |
|  | **1** | **2** | **3** | **4** | **5** | **6** | **7** | **8** | **9** | **10** | **11** | **12** | **13** | **14** | **15** | **16** | **17** | **18** | **19** | **20** |  |
| **ELSA** | 5097 | 3833 | 3221 | 2692 | 2362 | 1998 | 1582 | - | - | - | - | - | - | - | - | - | - | - | - | - |  |
| **HRS** | 8234 | 6814 | 5813 | 4728 | 3959 | 3262 | 2682 | 1954 | 1547 | 1138 | - | - | - | - | - | - | - | - | - | - |  |
| **InCHIANTI** | 1132 | 893 | 794 | 642 | - | - | - | - | - | - | - | - | - | - | - | - | - | - | - | - |  |
| **LASA** | 1742 | 1441 | 1135 | 793 | 580 | 408 | - | - | - | - | - | - | - | - | - | - | - | - | - | - |  |
| **MAP** | 1738 | 1529 | 1338 | 1209 | 1048 | 899 | 788 | 652 | 525 | 419 | 338 | 283 | 232 | 157 | 93 | 47 | 20 | 18 | 12 | 14 |  |

1. ELSA, English Longitudinal Cohort Study; HRS, Health and Retirement Study; InCHIANTI, InCHIANTI, Invecchiare in Chianti Study; LASA, Longitudinal Aging Study Amsterdam; MAP, Rush Memory and Aging Project.

**Table 7.** BIC Indices for Quadratic and Linear FI trajectory models

| **Cohort** | **BIC** | | | |
| --- | --- | --- | --- | --- |
|  | **Model 1** | **Model 2** | **Model 3** | **Model 4** |
|  | Linear | Linear with Sex/Gender*Age | Quadratic | Quadratic with Sex/Gender*Age |
| **ELSA** | -33951.239 | -33936.355 | -14391.657 | -14368.096 |
| **HRS** | -52033.699 | -52024.992 | -19080.273 | -19067.105 |
| **InCHIANTI** | -6086.584 | -6076.131 | -1447.846 | -1431.678 |
| **LASA** | -10984.466 | -10980.198 | -2233.577 | -2229.709 |
| **MAP** | -10863.790 | -22856.210 | -1670.200 | -13663.637 |

1. BIC, Bayesian Information Criterion.
